# Femtosecond-Resolved Excited State Relaxation Dynamics of Copper (II) Tetraphenylporphyrin (CuTPP) After Soret Band Excitation

**DOI:** 10.1038/s41598-017-17296-z

**Published:** 2017-12-04

**Authors:** Dahyi Jeong, Dong-gu Kang, Taiha Joo, Sang Kyu Kim

**Affiliations:** 1 Department of Chemistry, KAIST, Daejeon, 34141 Republic of Korea; 2 Department of Chemistry, POSTECH, Pohang, 37673 Republic of Korea

## Abstract

Excited state relaxation dynamics of Copper (II) tetraphenylporphyrin (CuTPP) after Soret band excitation have been investigated in various solvents by femtosecond broadband transient absorption spectroscopy. Significant role of charge transfer state has been confirmed from fast relaxation of triplet CuTPP in pyridine, giving τ ~ 26.5 ps. In piperidine, the transient measured at 480 nm shows biexponential behavior with distinct time constants of 300 fs and 27.4 ps. The fast component with τ ~ 300 fs is attributed to relaxation of the CuTPP-piperidine adduct populated in the ground state, giving the intrinsic relaxation rate of the CuTPP exciplex for the first time. For CuTPP in O-coordinating solvents of 1,4-dioxane and tetrahydrofuran (THF), a completely new relaxation channel via the ^2^[d_z2_, d_x2−y2_] state is opened. As the exciplex formation is diffusion controlled, triplet CuTPP lifetimes in pure solvents employed here are all measured to be more or less same to give ~30 ps, whereas the ^2^[d_z2_, d_x2−y2_] exciplex formed by the ligation with O-coordinating solvents is found to relax much slowly to the ground state, giving lifetimes of ~360 and ~270 ps in 1,4-dioxane and THF, respectively.

## Introduction

Porphyrin is one of the most important pigments in natural and artificial light-harvesting systems^1–8^, and thus it is essential to understand how light absorbed by the porphyrin pigment is converted into energy via electron/proton transfer processes. Accordingly, porphyrin and its various derivatives with and without metals have been subjected to both extensive and intensive studies for many decades especially in terms of their photochemical and photophysical properties. Since porphyrin molecules are large and their physicochemical properties are strongly dependent on chemical environments, it has been a formidable task to get the detailed picture of the whole light-energy conversion process at the molecular level. Nevertheless, there have been tremendous advances in this endeavor, and fundamental properties of porphyrins are well understood so that applications of specially-designed porphyrin derivatives to organic devices in solar cells^9–12^ and phototransistors^13,14^ are considered to be quite promising.

Ultrafast relaxation dynamics have been quite intensively studied for free base (H_2_TPP)^15–21^ and metalloporphyrins coordinating Zn^22–24^, Cu^25–27^, or Ni^28–31^. Herein, we are focusing on time-resolved relaxation dynamics of copper tetraphenylporphyrin (CuTPP) after its Soret-band excitation in many different solvents. Relaxation dynamics of CuTPP are known to be strongly dependent on nature of the solvent. For instance, according to the report by Kim *et al*.^32^, relaxation of the triplet CuTPP into the ground state is quite slow and takes more than 10 ns in non-coordinating solvents such as benzene or toluene whereas it becomes enormously faster by three orders of magnitude in N-containing solvents to give the lifetimes shorter than 40 ps or 120 ps in pyridine or piperidine, respectively. The huge increase of the relaxation rate of the triplet CuTPP was attributed to opening of new relaxation channel via the intramolecular ligand-to-metal charge transfer (CT) state which is much stabilized by N-coordinating solvents. Meanwhile, another relaxation channel was suggested by Kruglik *et al*. for excited CuTPP dissolved in O-coordinating solvents^33^. It was proposed that the excited-state complex (exciplex) of CuTPP with O-coordinating solvent is dissipated by fast relaxation via a low lying metal localized ^2^[d_z2_, d_x2−y2_] state. And yet, as a matter of fact, exciplex dynamics of CuTPP in O-containing solvents has not been much investigated. Rather, dynamics of water-soluble copper (II) tetrakis 4-N-methylpyridyl porphyrin (Cu(TMpy-P4)) molecule had been subjected to detailed studies. Jeoung *et al*.^25^, for instance, reported that formation and decay of the exciplex of Cu(TMpy-P4) in water take place with time constants of ~1.2 and ~4.0 ps, respectively. Significant role of the ^2^[d_z2_, d_x2−y2_] state in the same system had also been emphasized by Chirvony *et al*.^34^.

Herein, we aim to the better understanding of the relaxation mechanism of CuTPP in N- or O-coordinating solvents. We used the technique of femtosecond broadband transient absorption (TA) spectroscopy to systematically investigate fast relaxation processes of CuTPP in various solvent molecules after Soret band excitation. Dynamic roles of charge transfer and/or ^2^[d_z2_, d_x2−y2_] state are discussed with precisely measured time constants involved in the whole relaxation pathway. In this work, we provide complete TA spectra of CuTPP in various solvents as a function of the relaxation time with femtosecond time resolution for the first time.

## Result and Discussion

**Figure 1:**
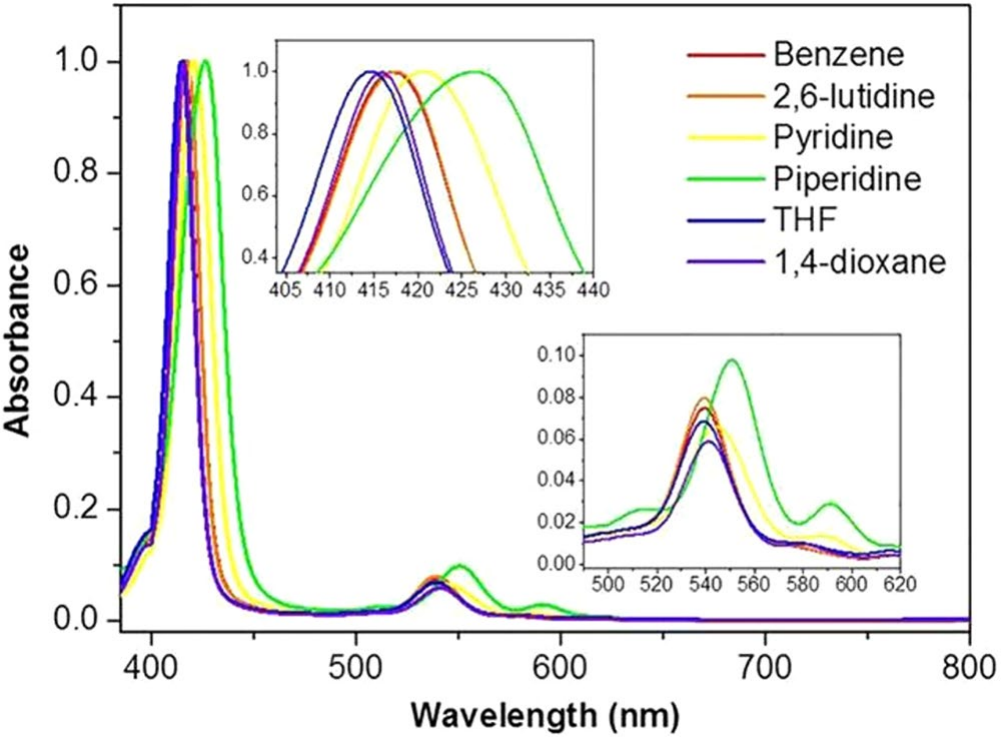
Normalized (in the arbitrary scale) absorption spectra of CuTPP in various solvents. The excitation wavelength for transient absorption experiment was fixed at 400 nm. Sample concentrations were ~10^−5^ M (Insets show enlarged absorption features near Soret and Q-bands).

Steady state absorption spectra of CuTPP show Soret and Q-bands in the UV and visible region, respectively (Fig. 1). Since interaction between copper ion and solvent molecules influences charge density of porphyrin ring in both ground and excited states, red- or blue-shift of the Soret band peak position compared to that in benzene is clearly observed for CuTPP in N- or O-coordinating solvents, respectively. Notably, red-shift of the Soret band is found to be quite large for CuTPP in piperidine. This could be attributed to solvent ligation of the ground-state CuTPP due to strong nucleophilicity of piperidine (*vide infra*)^32,35^. It is also noteworthy that Q(1,0) band is red-shifted by ~12 nm while oscillator strengths of Q(2,0) at 515 nm and Q(0,0) bands at 591 nm are much enhanced for CuTPP in piperidine compared to those in benzene. Fluorescence of CuTPP in the visible region is not observable because of the short lifetime of the S_1_ state^36^. All absorption bands of H_2_TPP and CuTPP with their assignments are summarized in Figure S1 and Table S1.

**Figure 2:**
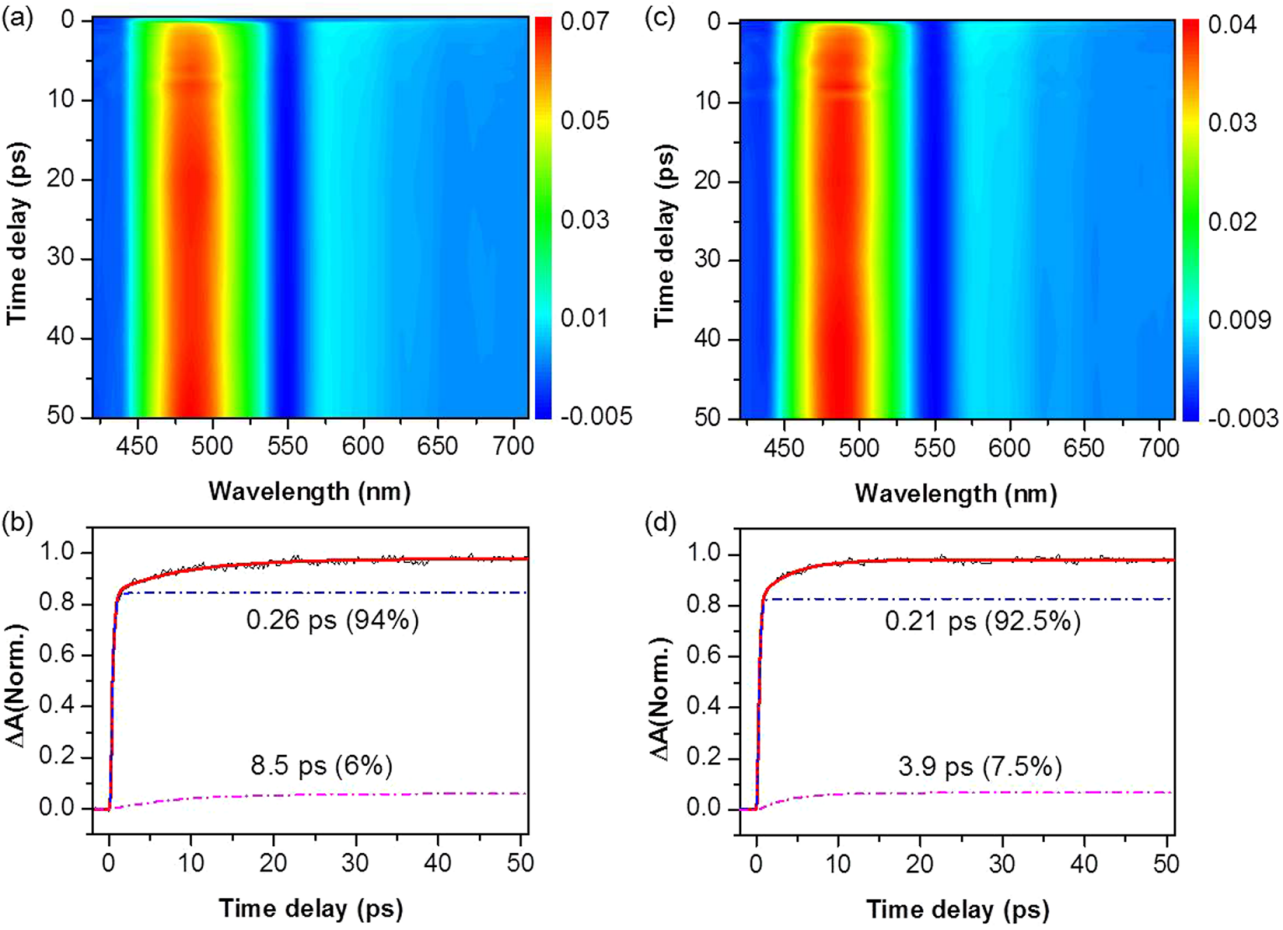
TA spectra of CuTPP in benzene (**a**,**b**) and 2,6-lutidine (**c**,**d**). (**a**,**c**) Contour plot of experimental data. (**b**,**d**) kinetic trace recorded at 480 nm wavelength. The kinetic traces were deconvoluted by two rise components corresponding to ISC from S_1_ to T_1_ (0.26 ps for benzene and 0.21 ps for 2,6-lutidine) and vibrational relaxation in T_1_ state (8.5 ps for benzene and 3.9 ps for 2,6-lutidine).

TA spectra of CuTPP after 400 nm excitation taken in non-coordinating solvents of benzene and 2,6-lutidine are shown in Fig. 2. It should be noted that 2,6-lutidine, despite its strong nucleophilicity, is not a coordinating solvent due to steric hindrance caused by the two methyl groups adjacent to nitrogen atom. Excited-state absorption (ESA) spectral pattern of CuTPP is quite different from that of H_2_TPP (Figure S4). Because the intersystem crossing (ISC) rate of 8.3 × 10^7^ s^−1^ measured for H_2_TPP is very slow^15^, the ESA signal of H_2_TPP manifested at early times represents dynamics of S_1_ which has been populated by ultrafast internal conversion (τ ~ 68 fs) from S_2_^27^. This is also quite consistent with high fluorescence quantum yield of H_2_TPP (0.11)^37,38^ which contrasts with little fluorescence from CuTPP (*vide supra*). The ESA signal of CuTPP reflects the triplet state dynamics. ESA intensities taken at 480 nm show biexponential rises with time constants of 0.26/8.5 ps in benzene and 0.21/3.9 ps in 2,6-lutidine. Ultrashort time constants then correspond to the population growth of T_1_ state by ISC from S_1_ while the longer time constants may be ascribed to vibrational relaxation in T_1_ state. Sub-picosecond ISC measured here is quite consistent with the gas-phase experimental result of 350 fs^26^. Slight blue-shift of transient absorption spectra has been observed for CuTPP in 2,6-lutidine in the similar time scale of vibrational cooling, supporting our assignment for slow-rising components. Time-dependent spectral characteristics, however, are not dramatic possibly due to interplay between vibrational cooling and solvation dynamics.

A positive correlation between vibrational cooling rate and polarity of solvent is well documented for many other systems^36,39–42^, and the faster vibrational relaxation of CuTPP (T_1_) in 2,6-lutidine (ε = 7.33) compared to that in benzene (ε = 2.27) could be rationalized accordingly.

**Figure 3:**
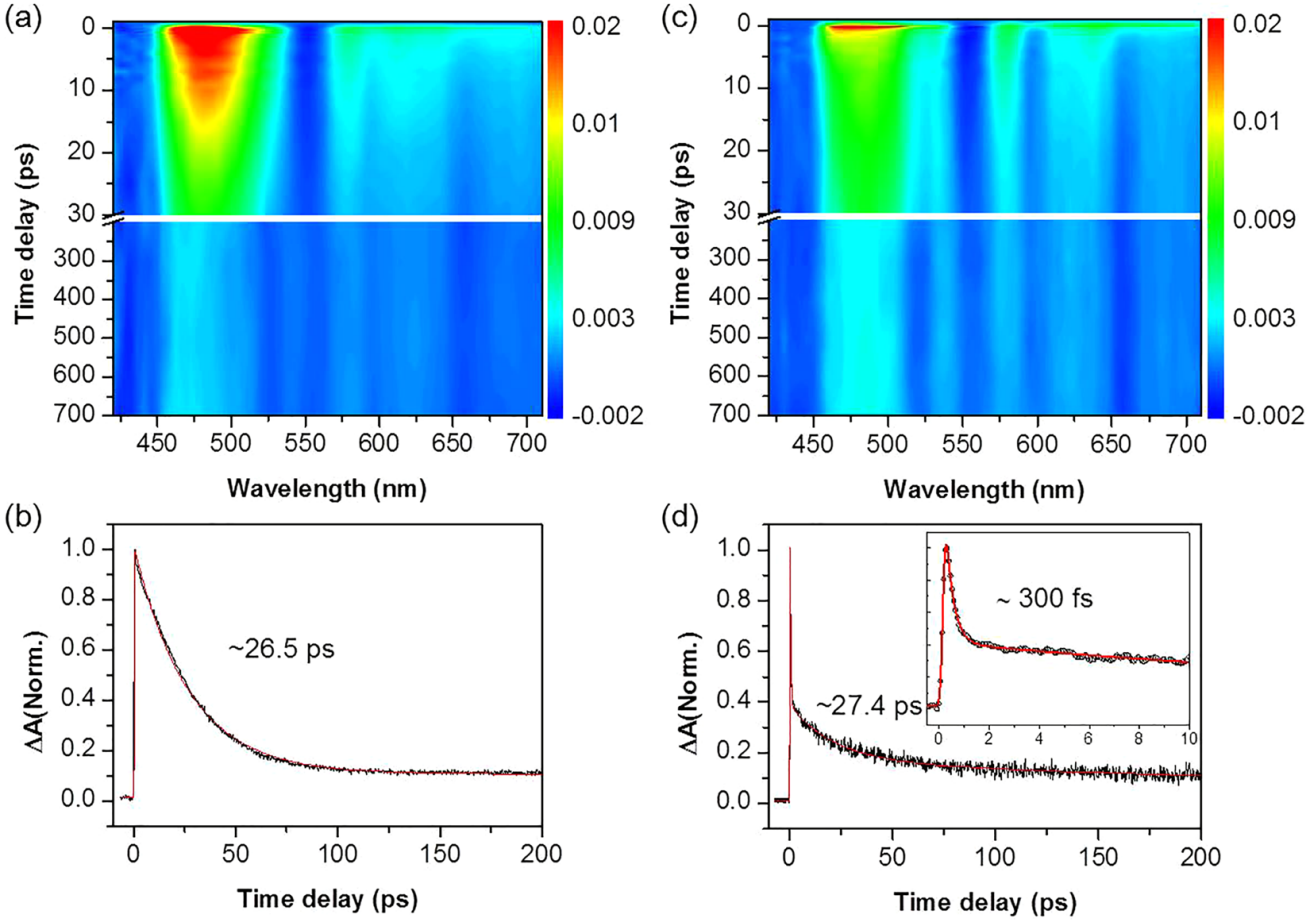
TA spectra of CuTPP in pyridine (**a**,**b**) and piperidine (**c**,**d**). (**a**,**c**) Contour plot of experimental data, (**b**,**d**) Kinetic traces recorded at 480 nm represented to triplet state relaxation. Ultrafast component observed in piperidine with time constant of 300 fs (inset in (**d**)) reflected the intrinsic lifetime of CuTPP-piperidine exciplex (see the text).

Excited-state relaxation dynamics of CuTPP in pyridine is remarkably different from those in non-coordinating solvents. For instance, as shown in Fig. 3, the 480 nm transient of CuTPP in pyridine shows instrument-limited ultrafast rise with the time constant shorter than 100 fs followed by single-exponential decay with a time constant of 26.5 ps. This indicates that ISC of CuTPP in pyridine is slightly faster than that in non-coordinating solvents and the triplet state relaxes quite rapidly in pyridine. It should be noted here that although the vibrational cooling process within the triplet manifold should be present in all solvents, it was observed only in benzene and 2,6-lutidine where the TA spectra show little change. Because the vibrational cooling process induces a minor spectral change, it was not discernable in other coordinating solvents, where the spectral dynamics are extensive because of the multifaceted relaxation channels including diffusion-limited relaxation process by solvent-coordination. To interrogate the role of pyridine in the triplet quenching, we have obtained the 480 nm transient as a function of the pyridine concentration in benzene from 1.24 M to 12.4 M (Figure S5). Triplet quenching is much slowed down as the pyridine concentration decreases, clearly showing the essential role of the fifth coordination of pyridine to the copper ion in efficient triplet state relaxation. Triplet CuTPP relaxation rate is found to be proportional to the pyridine concentration with the rate constant of k ~ 2.1 × 10^9^ M^−1^ S^−1^. This indicates that triplet state relaxation is prompt once CuTPP forms the exciplex with pyridine, demonstrating that this process is diffusion controlled. The metal-to-ligand charge transfer state which is largely stabilized by solvent ligation is believed to play a significant role in facilitating the excited-state dissipation. Our relaxation time constant of 26.5 ps for the triplet CuTPP in pyridine is the most precise value up to date while it is consistent with the previous result by Kim *et al*. in which its upper limit was reported to be ~40 ps^32^.

Relaxation dynamics of CuTPP in piperidine, another N-coordinating solvent, is supposed to be not much different from that in pyridine. Accordingly, the 480 nm transient of CuTPP in piperidine (Fig. 3(d)) also shows the instrument-limited fast rise due to ultrafast ISC. In contrast to the TA signal in pyridine, however, the 480 nm transient of CuTPP in piperidine shows bi-exponential decay with two distinct time constants of 300 fs and 27.4 ps. The longer time constant in piperidine is quite close to the decay time (26.5 ps) observed in pyridine, and thus kinetics of this component may represent the rate of the exciplex formation by solvent diffusion. The triplet-state lifetime of 27.4 ps for CuTPP in piperidine here is much shorter than the previously reported value of 120 ps^32^ while the fast component (~300 fs) had not been observed in any other previous studies. This should be due to that temporal resolution of other previous studies was not high enough for observation of kinetic components shorter than a picosecond. It should be reminded that there is a large solvent-induced red-spectral shift of CuTPP in piperidine (*vide supra*). This indicates that significant portion of CuTPP in piperidine, due to its strong nucleophilicity, exists as the five-coordinated complex in the ground state^32^. Actually, according to Sankar *et al*.^35^, about 40% of CuTPP in piperidine exist as the CuTPP:solvent adduct in the ground state while only 3% is measured to be present as an adduct in pyridine. In this aspect, the time constant of 300 fs may represent relaxation of the excited species which are prepared by Soret band excitation of the CuTPP-piperidine adduct which is already populated in the ground state. For diffusion-controlled processes, experimentally measured lifetimes are mainly associated with the rate of translational and/or rotational diffusion of solvent. Time constant measured in this environment thus does not provide intrinsic lifetime of the excited state. As the CuTPP-piperidine adduct is significantly populated in the ground state, we could identify and estimate the intrinsic relaxation property of the CuTPP-piperidine complex without being interfered by the solvent diffusion rate for the first time. Indeed, the intrinsic relaxation rate of the five-coordinated complex is found to be very fast (~300 fs), and we expect that this rate would be more or less same in pyridine and piperidine as long as the solvent ligation to the triplet CuTPP is ensured. This once again confirms that the CT exciplex, once solvent ligated, relaxes to the ground state very rapidly, which is in accord with the main concept of diffusion controlled process.

**Figure 4:**
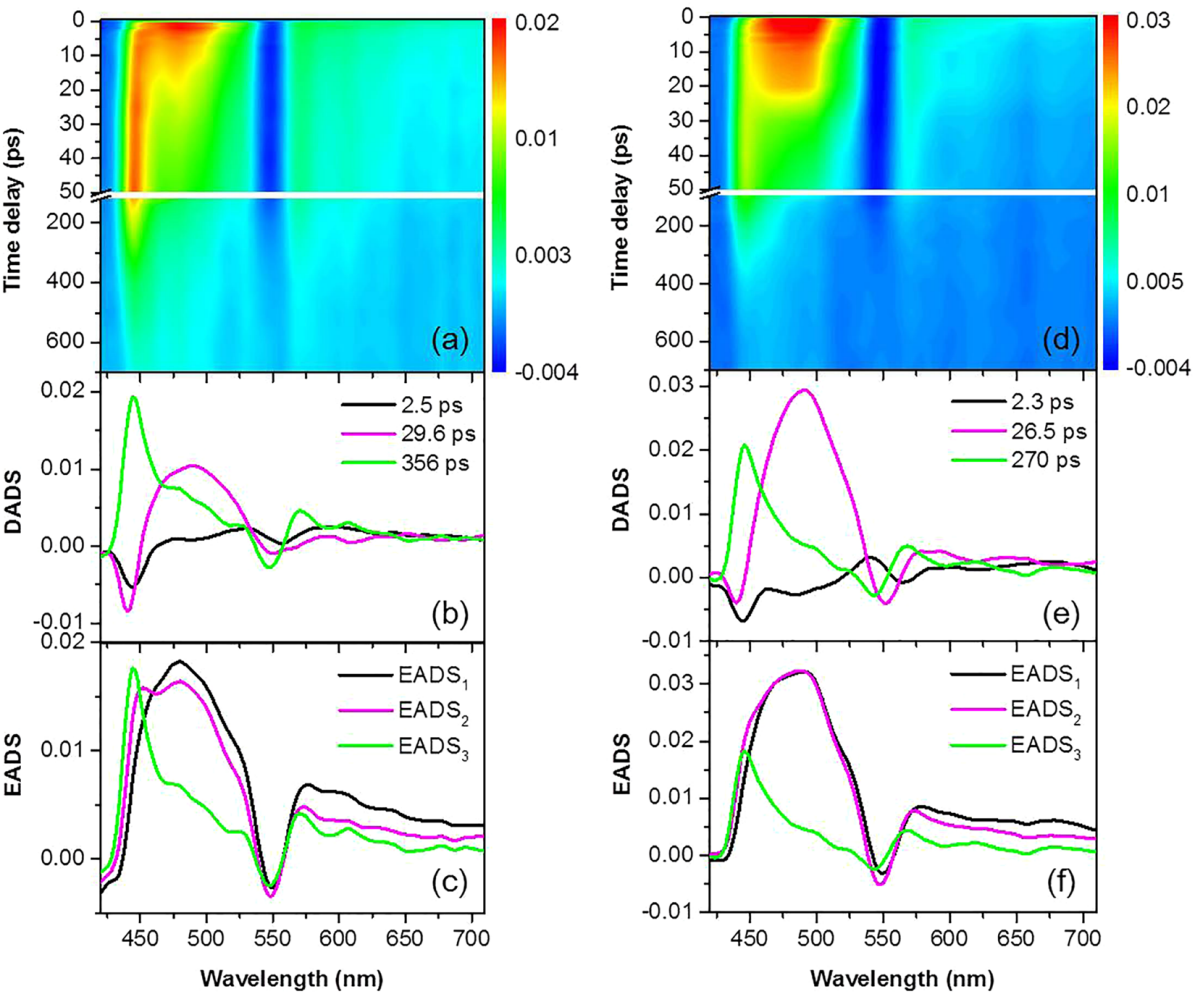
TA spectra and global analysis of CuTPP in 1,4-dioxane (**a**–**c**) and THF (**d**–**f**) after 400 nm excitation. (**a**,**d**) Contour plot of the experimental data, (**b**,**e**) DADS of TA spectra and (c, f) EADS of TA spectra are shown. Unit for ordinate is ΔA in DADS and EADS.

TA spectra of CuTPP in O-coordinating solvents such as 1,4-dioxane and THF are found to be quite different from those in N-coordinating solvents. As shown in Fig. 4, a strong and narrow ESA band centered at ~440 nm is newly observed, while the broad ESA signal in the 450–490 nm regions remains similar to that observed in N-coordinating solvents. The 440 nm band grows as the broad band over the 450–490 nm decays. In order to extract individual kinetic components from TA spectra, global analysis of decay associated difference spectra (DADS) and evolution associated difference spectra (EADS) have been performed by using the Glotaran (1.5.1) program. Prior to global analysis, a singular value decomposition (SVD) procedure was applied to estimate how many individual components exist in the TA spectra. As shown in Fig. 4, three independent components have been identified in TA spectra of CuTPP in 1,4-dioxane and THF. Although small in amplitudes, the first 2.5 ps component (black line in Fig. 4(b)) of DADS of CuTPP in 1,4-dioxane shows ESA around 480 nm and ground state bleach at 550 nm, which are reminiscent of the TA spectrum of CuTPP observed in non-coordinating and N-coordinating solvents at early times. Additionally, a rising band at 440 nm is newly observed at 2.5 ps. The second 29.6 ps component (magenta line in Fig. 4(b)) shows the decay of the broad 450–530 nm band and increase of the 440 nm band. Temporal behavior of the 450–530 nm ESA band decaying with time constant of 29.6 ps is very similar to that observed for CuTPP in N-coordinating solvents in terms of both temporal and spectral characteristics. In this regard, this decay could be attributed to relaxation of the triplet CuTPP via solvent ligation through the CT state. On the other hand, the 29.6 and 356 ps DADS components shows a clear rise and fall of the relatively sharp 440 nm ESA band, respectively. Therefore, the 440 nm band, which is uniquely observed in O-coordinating solvents, behaves very differently; it rises by two time constants 2.5 and 29.6 ps and decays with a much longer time constant of 356 ps. This result is consistent with the EADS analysis, which is based on a sequential model with increasing lifetimes. For the TA spectra of CuTPP in 1,4-dioxane, the 440 nm feature is the most prominent in EADS_3_ indicating that it rises mostly by 29.6 ps and in part 2.5 ps. The same is true in THF, although amplitude of the rise is smaller than that in 1,4-dioxane. The integrated time traces near the 440 nm band also show the biexponential rise features which are comparable with the time constants obtained from the global analysis (Figure S6).

As both spectral and temporal dynamics of this rather sharp 440 nm band are distinct from those of the broad 450–530 nm band, this experimental fact clearly indicates that there is another relaxation pathway in O-coordinating solvents. The most plausible one is the relaxation pathway via the metal localized ^2^[d_z2_, d_x2−y2_] state which was proposed earlier by Kruglik *et al*. from their resonance Raman and picosecond absorption spectroscopic study^33^. From our result, the triplet CuTPP in 1,4-dioxane may then have two different relaxation pathways. One is the relaxation of the triplet CuTPP via the metal-to-ligand charge transfer state stabilized by the exciplex formation with solvent, similarly to the case of CuTPP relaxation in N-coordinating solvents. The other channel, which is opened only in O-coordinating solvents, is the relaxation via the near-lying ^2^[d_z2_, d_x2−y2_] state. Presumably, the ^2^[d_z2_, d_x2−y2_] state becomes energetically much lowered by coordination of the O-coordinating solvent so that this new relaxation channel becomes as efficient as the relaxation via the CT state. The experimental finding that the sharp 440 nm band grows concurrently as the broad band decays suggests that the 440 nm band represents population of the ^2^[d_z2_, d_x2−y2_] exciplex. Formation of the ^2^[d_z2_, d_x2−y2_] exciplex is expected to be also diffusion limited, and thus the rising time constant of 29.6 ps for the sharp 440 nm band is well correlated with the lifetime of the triplet CuTPP manifested by the decay of the broad ESA band. The smaller amplitude 2.5 ps rise component could indicate that some of the CuTPP in O-coordinating solvent also forms adduct in the ground state to form the exciplex rapidly although it is subject to further studies. The decay of the 440 nm band indicates that the ^2^[d_z2_, d_x2−y2_] exciplex in 1,4-dioxane disappears with τ ~ 360 ps. This is much slower than the CT exciplex lifetime of 300 fs in piperidine (*vide supra*).

Simple kinetic scheme regarding relaxation of the triplet CuTPP in O-coordinating solvent can be then described as follows.

$${}^{2}{\rm{S}}_{2}\to {}^{2}{\rm{S}}_{1}\to {}^{2}{\rm{T}}_{1}+{\rm{L}}\to {{\rm{T}}}_{1}{({\rm{L}})}_{d,d}({\rm{or}}\,{{\rm{T}}}_{1}{({\rm{L}})}_{{\rm{CT}}})\to {}^{2}{\rm{S}}_{0}$$

Here internal conversion from S_2_ to S_1_ is extremely fast (τ ~ 68 fs)^27^ and ISC from S_1_ to T_1_ takes place in sub-picosecond time scale (*vide supra*). Therefore, according to this, the rise of the 440 nm band with τ ~ 29.6 ps should represent the population growth of the ^2^[d_z2_, d_x2−y2_] exciplex. This is identical to the decay of the broad 450–530 nm band, which should be associated with the rate of solvent diffusion. Indeed, time constant of ~30 ps measured in 1,4-dioxane is more or less same as the lifetime of the triplet CuTPP in N-coordinating solvent. Meanwhile, decay of the 440 nm band should represent the relaxation rate of the ^2^[d_z2_, d_x2−y2_] exciplex to the ground electronic state. It is quite surprising that the relaxation rate of the ^2^[d_z2_, d_x2−y2_] exciplex is one order of magnitude slower than the relaxation rate via the CT exciplex. The relatively narrow spectral width of the ESA band of the ^2^[d_z2_, d_x2−y2_] state may be responsible for the slower relaxation dynamics as the narrow spectral window could be the consequence from the structural rigidity during the relaxation process, resulting in the decrease of statistical number of different ways of relaxation. As two distinct CT and ^2^[d_z2_, d_x2−y2_] relaxation pathways compete, the ratio of the two different processes should be strongly dependent on the detailed thermodynamic energetics involved in the exciplex formation by solvent ligation. Naturally, one can expect that the relative ratio of CT to ^2^[d_z2_, d_x2−y2_] relaxation pathways depends on the physical property of the solvent. From species associated difference spectra (SADS) target analysis (Figure S7), it is estimated that approximately 32% of the triplet CuTPP decays via the CT state in THF, whereas only 4% is found to decay via the CT state in 1,4-dioxane. These branching ratios are comparable to the relative intensities of the sharp 440 nm band in TA spectra (Fig. 4(a),(d)). Namely, qualitatively speaking, the triplet CuTPP, once ligated with 1,4-dioxane, mainly relaxes via the ^2^[d_z2_, d_x2−y2_] state whereas some portion relaxes into the ground state via the CT state. While the lifetime of the triplet CuTPP remains constant for both O-coordinating solvents, the relaxation rate of the ^2^[d_z2_, d_x2−y2_] exciplex is found to be faster in THF, giving τ ~ 270 ps compared to 360 ps in 1,4-dioxane. The origin of this difference is not clear at the present time. One plausible scenario is that the ^2^[d_z2_, d_x2−y2_] exciplex in THF is energetically remote from the triplet CuTPP compared to that in 1,4-dioxane.

Herein, excited state relaxation dynamics of CuTPP in various solvents have been interrogated in detail using the femtosecond broadband TA spectroscopic technique. Relaxation dynamics of the triplet CuTPP is found to be strongly dependent on whether or not the surrounding solvent molecule could coordinate to the copper ion. Lifetime of the triplet CuTPP is more than tens of nanosecond in non-coordinating solvents such as benzene or 2,6-lutidine. It decreases tremendously in coordinating solvents such as N-coordinating pyridine and piperidine or O-coordinating 1,4-dioxane and THF. Coordination kinetics of pyridine to CuTPP are found to be diffusion-limited, confirming that the triplet CuTPP exciplex formed by solvent ligation plays a major role in facilitating relaxation to the ground state. In piperidine, the transient shows biexponential behavior with distinct time constants of 300 fs and 27.4 ps. The newly found fast component with τ ~ 300 fs is most likely due to the relaxation of the CuTPP-piperidine adduct which is formed in the ground state, providing the intrinsic relaxation rate of the CuTPP-exciplex for the first time. This is the first evidence for the fast relaxation of the charge transfer complex of triplet CuTPP in N-coordinating solvent. For CuTPP in O-coordinating solvents such as 1,4-dioxane and THF, we have found that there should exist a new relaxation channel in addition to the charge transfer relaxation pathway, and these two different relaxation channels compete with each other. The new channel is here ascribed to relaxation via the ^2^[d_z2_, d_x2−y2_] exciplex^33^. Our comprehensive TA data reveal, for the first time, detailed quantitative dynamics of these two competing channels involved in the triplet CuTPP relaxation. Briefly, the triplet CuTPP converts to the CT exciplex through solvent ligation in N-coordinating solvents, whereas it goes to either CT or ^2^[d_z2_, d_x2−y2_] exciplex in O-coordinating solvents with τ ~ 30 ps. As the exciplex formation is diffusion controlled, triplet CuTPP lifetimes in all solvents used here are measured to be more or less the same. The intrinsic relaxation rate of the CT exciplex in piperidine is measured to be very fast (τ ~ 300 fs), whereas the ^2^[d_z2_, d_x2−y2_] exciplex is found to relax much slowly to the ground state, giving lifetimes of ~360 ps or ~270 ps in 1,4-dioxane or THF, respectively.

## Methods

### Materials and Solvents

**Figure 5:**
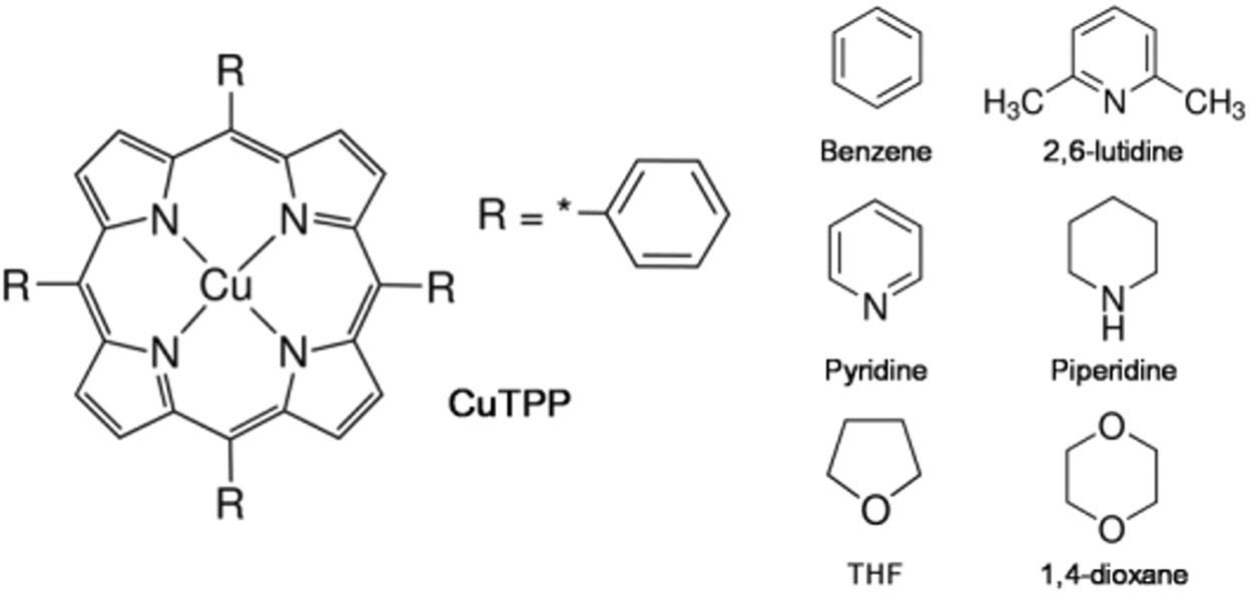
The molecular structure of CuTPP and various solvents used in experiments.

5,10,15,20-Tetraphenyl-21*H*,23*H*-porphine (≥99%; H_2_TPP) and 5,10,15,20-Tetraphenyl-21*H*,23*H*-porphine copper (II) (99.5%; CuTPP) were purchased from Sigma-Aldrich and dissolved in various solvents to get solutions of concentrations in the 5 × 10^−5^–1 × 10^−4^ M range without further purification. As depicted in the Fig. 5, non-coordinating solvents (benzene, 2,6-lutidine), N-coordinating solvents (pyridine, piperidine), and O-coordinating solvents (THF, 1,4-dioxane) were used.

### Steady-state measurements and Time-resolved fluorescence spectra

UV-vis absorption and emission spectra were obtained by using the V0530 UV-VIS (Jasco) and RF-5301PC fluorescence spectrometer (Shimadzu), respectively, and fluorescence lifetimes were measured by using the time-correlated single-photon counting (TCSPC) method (FL920, Edinburgh Instruments).

### Femtosecond transient absorption spectroscopy

Femtosecond laser pulse at 800 nm with the temporal width of ~35 fs and 1 kHz repetition rate (Vitara-S and Legend Elite USP, Coherent) was split into two pulses using a 3:1 beam splitter. The laser pulse for pumping was frequency doubled through a BBO crystal (200 μm thick) to generate the 400 nm output. The other beam was focused on a 3 mm thick linearly moving CaF_2_ window to produce white-light continuum (WLC) in the spectral range of 350–750 nm. The WLC output was split again into reference and probe pulses where the reference pulse was used for shot-to-shot normalization of the fluctuating probe pulse intensity. Each CuTPP solution was loaded in a quartz cell with a 1 mm path length. The quartz cell was continuously moved back and forth in the perpendicular direction to the propagation axis of the laser pulse to minimize photo-damage of samples. Pump and probe laser pulses were spatially overlapped in the middle of the sample cell. After passing through the cell, reference and probe pulses were focused on entrance slit of a monochromator (Monora200, Dongwoo-Optron) to be detected by Si pre-amplified photodetectors (2032, Newfocus) as a function of the wavelength. Difference between probe laser intensities with and without pump laser pulse was monitored using a lock-in amplifier (SR810, Stanford Research System) coupled with a chopper. Group velocity dispersion (GVD) of the WLC was compensated by measuring time-zero positions at different wavelengths. Using the optical Kerr effect (OKE) from hexanes, time-zero positions where pump and probe laser pulses are temporally overlapped were measured at many different wavelengths in the 400–700 nm region. This gave a polynomial fitting function by which the grating and optical-delay stage were controlled simultaneously to compensate the GVD effect (Figure S3). All data were obtained at the magic angle (54.7°) to remove any polarization-dependent anisotropic dynamics. To extract meaningful time constants from TA spectra, the decay-associated difference spectra (DADS), evolution-associated difference spectra (EADS) and species-associated difference spectra (SADS) were taken from global and target analyses using the software package Glotaran 1.5.1 (http://glotaran.org).

## Electronic supplementary material

Supplementary Information

## References

[1] Kuciauskas D (1999). An Artificial Photosynthetic Antenna-Reaction Center Complex. J. Am. Chem. Soc..

[2] Lin VSY, DiMagno SG, Therien MJ (1994). Highly conjugated, acetylenyl bridged porphyrins: New models for light-harvesting antenna systems. Science..

[3] Balzani V, Credi A, Venturi M (2008). Photochemical Conversion of Solar Energy. ChemSusChem..

[4] Imahori H (2004). Giant Multiporphyrin Array as Artificial Light-Harvesting Antennas. J. Phys. Chem. B..

[5] Kay A, Graetzel M (1993). Artificial photosynthesis. 1. Photosensitization of titania solar cells with chlorophyll derivatives and related natural porphyrins. J. Phys. Chem..

[6] D’Souza F (2004). Energy Transfer Followed by Electron Transfer in a Supramolecular Triad Composed of Boron Dipyrrin, Zinc Porphyrin, and Fullerene: A Model for the Photosynthetic Antenna-Reaction Center Complex. J. Am. Chem. Soc..

[7] Imahori H, Umeyama T, Ito S (2009). Large π-Aromatic Molecular as Potential Sensitizers for Highly Efficient Dye-Sensitized Solar Cells. Acc. Chem. Res..

[8] Kodis G (2002). Efficient Energy Transfer and Electron Transfer in an Artificial Photosynthetic Antenna-Reaction Center Complex. J. Phys. Chem. A.

[9] Li L-L, Diau EW (2013). –G. Porphyrin-sensitized solar cells. Chem. Soc. Rev..

[10] Chae SH (2014). Novel π-extended porphyrin derivatives for use in dye-sensitized solar cells. J. Porphyrins Phthalocyanines.

[11] Mathew S (2014). Dye-sensitized solar cells with 13% efficiency achieved through the molecular engineering of porphyrin sensitizers. Nat. Chem..

[12] Martinez-Diaz MV, Torre Gdela, Torres T (2010). Lighting porphyrins and phthalocyanines for molecular photovoltaics. Chem. Commun..

[13] Seol M-L (2012). Hybrid Porphyrin-Silicon Nanowire Field-Effect Transistor by Opto-Electrical Excitation. ACS Nano.

[14] Choi S (2013). Dramatic enhancement of carrier mobility via effective secondary structural arrangement resulting from the substituents in a porphyrin transistor. Chem. Commun..

[15] Baskin JS, Yu H-Z, Zewail AH (2002). Ultrafast Dynamics of Porphyrins in the Condensed Phase: I. Free Base Tetraphenylporphyrin. J. Phys. Chem. A.

[16] Kumar PH (2015). Ultrafast Relaxation Dynamics of 5,10,15,20-meso-Tetrakis Pentafluorophenyl Porphyrin Studied by Fluorescence Up-Conversion and Transient Absorption Spectroscopy. J. Phys. Chem. A.

[17] Białkowski B (2012). The dynamics and origin of the unrelaxed fluorescence of free-base tetraphenylporphyrin. J. Photochem. Photobio. A: Chem..

[18] Kim KS (2011). Origin of Ultrafast Radiationless Deactivation Dynamics of Free-Base Subpyriporphyrins. J. Phys. Chem. Lett..

[19] Venkatesh Y, Venkatesan M, Ramakrishna B, Bangal PR (2016). Ultrafast Time-Resolved Emission and Absorption Spectra of meso-Pyridyl Porphyrins upon Soret Band Excitation Studied by Fluorescence Up-Conversion and Transient Absorption Spectroscopy. J. Phys. Chem. B.

[20] Kim SY, Joo T (2015). Coherent Nuclear Wave Packets in Q States by Ultrafast Internal Conversions in Free Base Tetraphenylporphyrin. J. Phys. Chem. Lett..

[21] Marcelli A (2008). Excited-State Absorption and Ultrafast Relaxation Dynamics of Porphyrin, Diprotonated Porphyrin, and Tetraoxaporphyrin Dication. J. Phys. Chem. A.

[22] Lee S (2014). Excited-state electronic couplings in a 1,3-butadiyne-bridged Zn(II)porphyrin dimer and trimer. Chem. Commun..

[23] Yu H-Z, Baskin JS, Zewail AZ (2002). Ultrafast Dynamics of Porphyrins in the Condensed Phase: II. Zync Tetraphenylporphyrin. J. Phys. Chem. A.

[24] Banerji N (2011). Ultrafast excited-state dynamics of strongly coupled porphyrin / core-substituted-naphthalenediimide dyads. Phys. Chem. Chem. Phys..

[25] Jeoung SC, Takeuchi S, Tahara T, Kim D (1999). Ultrafast decay dynamics of photoexcited Cu(II)(TMpy-P4) in water solvent. Chem. Phys. Lett..

[26] Ha-Thi M-H (2013). An Efficient Indirect Mechanism for the Ultrafast Intersystem Crossing in Copper Porphyrins. J. Phys. Chem. A.

[27] Yeon KY, Jeong D, Kim SK (2010). Intrinsic lifetimes of the Soret bands of the free-base tetraphenylporphine (H_2_TPP) and Cu(II)TPP in the condensed phase. Chem. Commun..

[28] Eom HS (1997). Ultrafast Vibrational Relaxation and Ligand Photodissociation/Photoassociation Processes of Nickel(II) Porphyrins in the Condensed Phase. J. Phys. Chem. A.

[29] Retsek JL (2003). Photoinduced Axial Ligation and Deligation Dynamics of Nonplanar Nickel Dodecaarylporphyrins. J. Am. Chem. Soc..

[30] Rodriguez J, Holten D (1989). Ultrafast vibrational dynamics of a photoexcited metalloporphyrin. J. Chem. Phys..

[31] Zamyatin AV, Gusev AV, J. Rodgers MA (2004). Two-Pump-One-Probe Femtosecond Studies of Ni(II) Porphyrins Excited States. J. Am. Chem. Soc..

[32] Kim D, Holten D, Gouterman M (1984). Evidence from picosecond transient absorption and kinetics studies of charge-transfer states in copper(II) porphyrins. J. Am. Chem. Soc..

[33] Kruglik SG (1995). Resonance Raman, CARS, and Picosecond Absorption Spectroscopy of Copper Porphyrins: The Evidence for the Exciplex Formation with Oxygen-Containing Solvent Molecules. J. Phys. Chem..

[34] Chirvony VS, Négrerie M, Martin J-L, Turpin P-Y (2002). Picosecond Dynamics and Mechanisms of Photoexcited Cu(II)-5,10,15,20-meso-tetrakis(4-N-methylpyridyl)porphyrin Quenching by Oxygen-Containing Lewis-Base Solvents. J. Phys. Chem. A.

[35] Sankar M, Arunkumar C, Bhyrappa P (2004). Unusual solvent dependent electronic absorption spectral properties of nickel(II) and copper (II) perhaloporphyrins. J. Porphyrins Phthalocyanines.

[36] Liu F (1995). Luminescence Quenching of Copper(II) Porphyrins with Lewis Bases. Inorg. Chem..

[37] Ohno O, Kaizu Y, Kobayashi H (1985). Luminescence of some metalloporphyrins including the complexes of the IIIb group. J. Chem. Phys..

[38] Seybold PG, Gouterman M (1969). Porphyrins: XIII: Fluorescence spectra and quantum yields. J. Mol. Spectrosc..

[39] Billsten HH, Zigmantas D, Sundström V, Polívka T (2002). Dynamics of vibrational relaxation in the S_1_ state of carotenoids having 11 conjugated C=C bonds. Chem. Phys. Lett..

[40] Bonn M, Brugmans MJP, Bakker HJ (1996). Solvent-dependent vibratioinal relaxation pathways after successive resonant IR excitation to υ = 2. Chem. Phys. Lett..

[41] Kovalenko SA, Schanz R, Hennig H, Ernsting NP (2001). Cooling dynamics of an optically excited molecular probe in solutioin from femtosecond broadband transient absorption spectroscopy. J. Chem. Phys..

[42] Wilderen LJGW, van, Kern-Michler D, Muller-Werkmeister HM, Bredenbeck J (2014). Vibrational dynamics and solvatochromism of the label SCN in various solvents and haemoglobin by time dependent IR and 2D-IR spectroscopy. Phys. Chem. Chem. Phys..

