# Supplementary material for: Femtosecond-Resolved Excited State Relaxation Dynamics of Copper (II) Tetraphenylporphyrin (CuTPP) After Soret Band Excitation
Source: Sci Rep. 2017 Dec 4;7:16865. doi: 10.1038/s41598-017-17296-z (PMC5715150; doi:10.1038/s41598-017-17296-z)
Supplement: Supplementary file 1 — Supplementary Information [file 41598_2017_17296_MOESM1_ESM.pdf]

Supporting Information

Femtosecond-resolved excited state relaxation dynamics of Copper (Ⅱ) Tetraphenylporphyrin (CuTPP) after Soret band excitation

Dahyi Jeong, [†1] Dong-gu Kang, [†2] Taiha Joo* [‡] and Sang Kyu Kim* [†]

[†]*Department of Chemistry, KAIST, Daejeon 34141, Korea*

[‡]*Department of Chemistry, POSTECH, Pohang 37673, Korea*

[1,2]Contributed equally

Corresponding authors: thjoo@postech.ac.kr (T.J.), sangkyukim@kaist.ac.kr (S.K.K.)

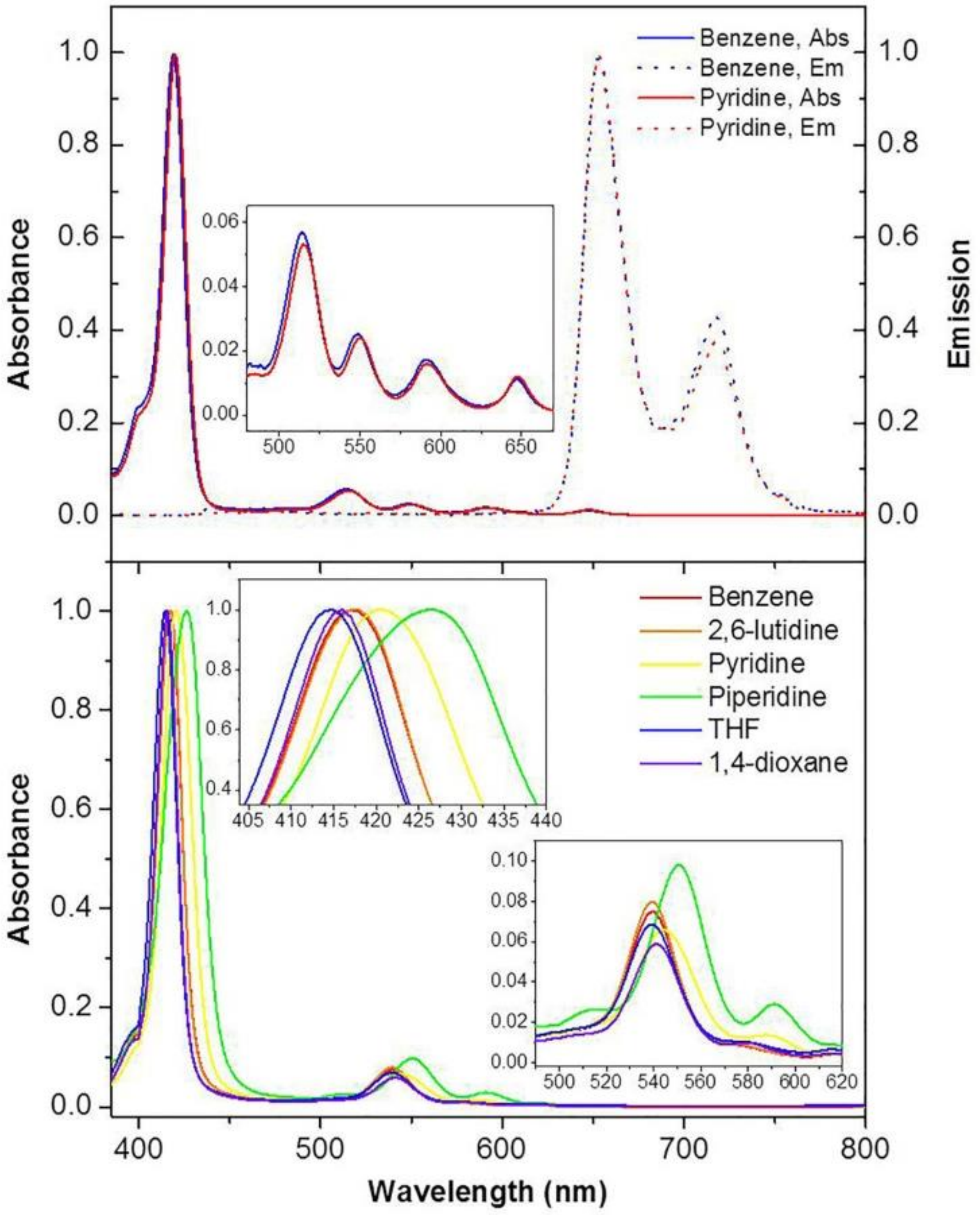

Figure S1. Normalized (in the arbitrary scale) absorption spectra of H_2TPP (Top) and CuTPP (Bottom) in various solvents. The excitation wavelength was fixed at 340 nm. Sample concentrations were ~ 10^{-5} M (Insets show enlarged absorption features near Soret and Q-bands).

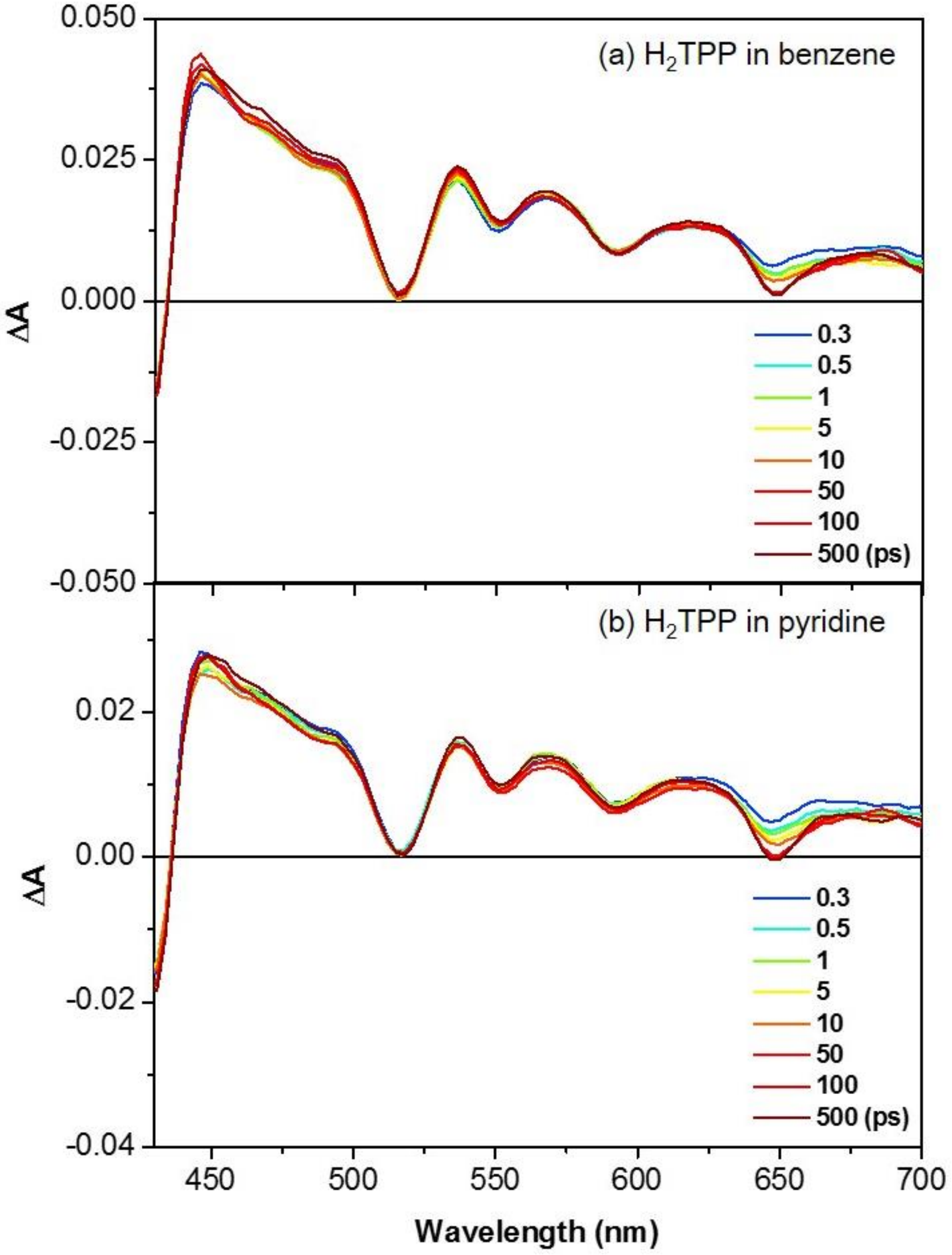

Figure S2. TA spectra of H_2TPP (a) in benzene and (b) pyridine after the 400 nm excitation.

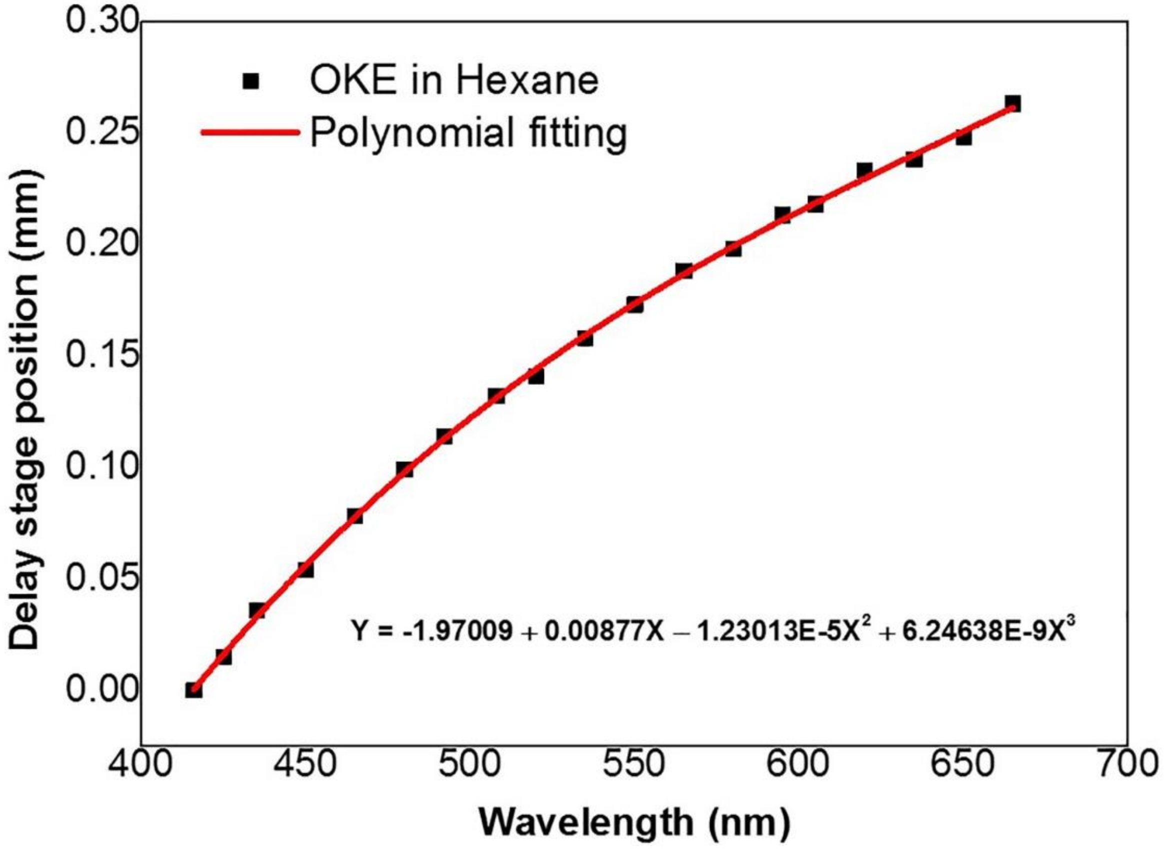

Figure S3. GVD compensation of WLC. The black square represents OKE signal obtained from hexane and the solid red line is its polynomial fit. The time resolution of delay stage is 6.67 fs/um. Our temporal spans correspond to ~2 ps in the range of WLC.

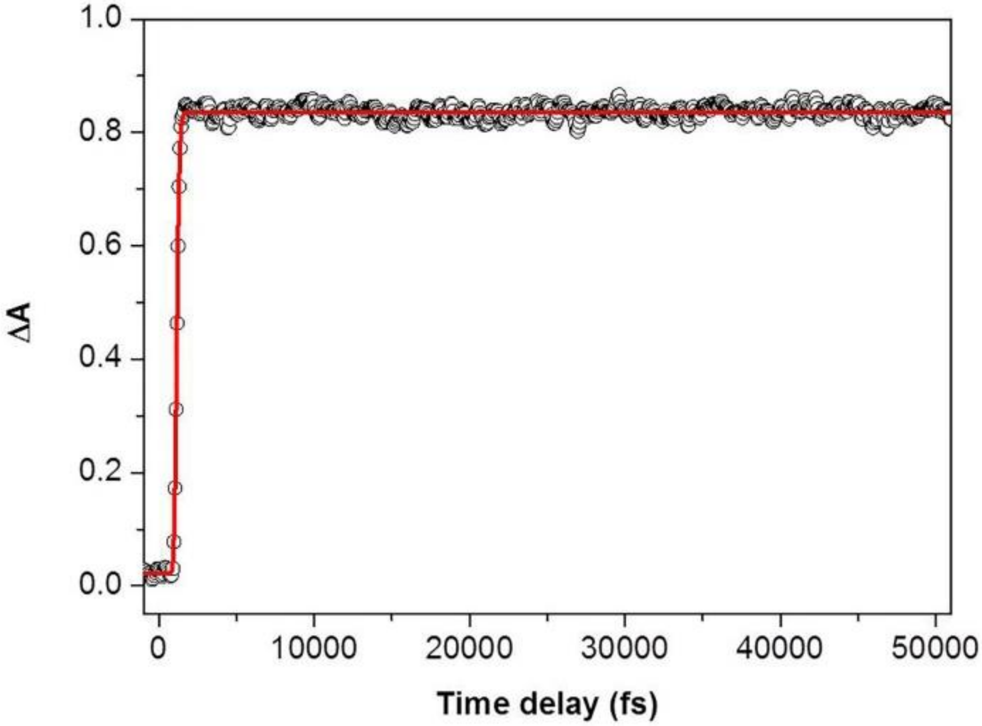

Figure S4. Transient of H_2TPP probed at 480 nm in benzene. The rising time constant of ~ 85 fs corresponds to the ultrafast internal conversion from S_2 state to S_1 state.

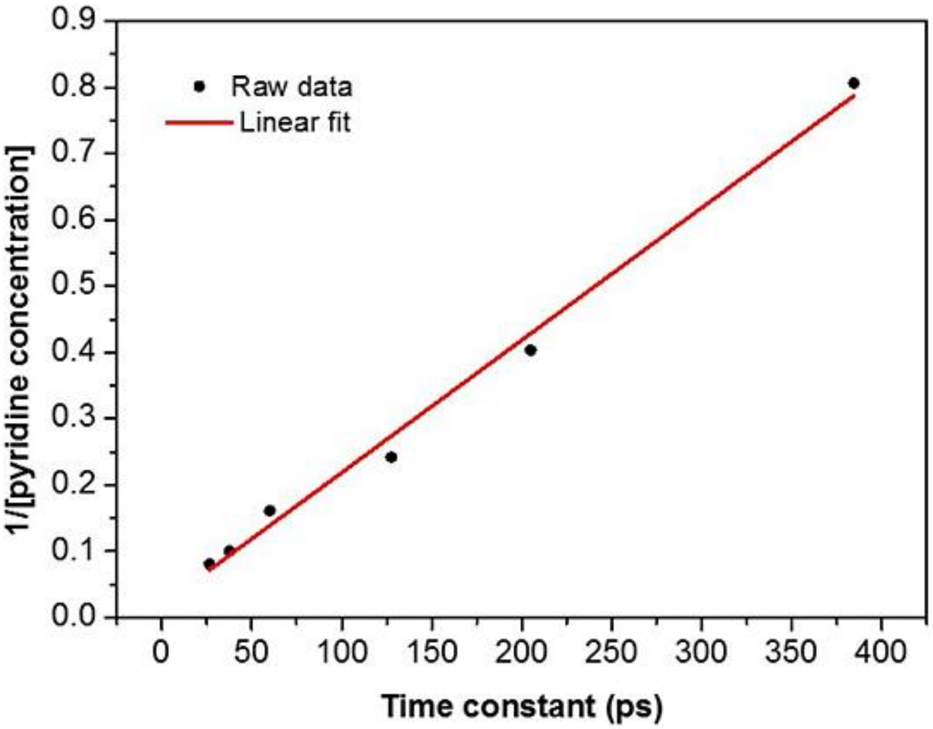

Figure S5. Second order kinetics for the solvent ligation of CuTPP showing the lifetime dependence on the pyridine concentration from 1.2 M to 12.4 M.

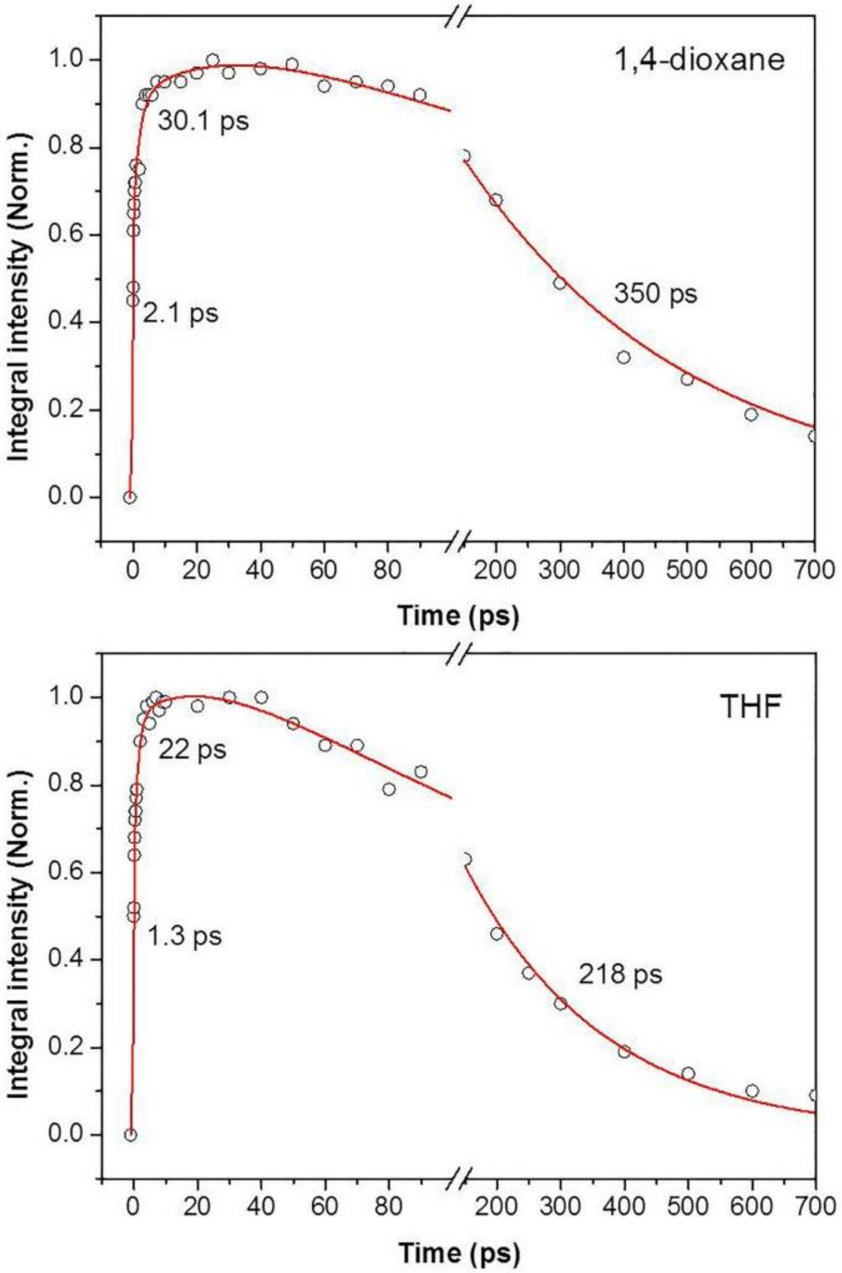

Figure S6. Integrated time traces near the 440 nm band in O-coordinating solvents. Two rise time constants (1-2 ps and 20-30 ps) are comparable with the values obtained from global analysis.

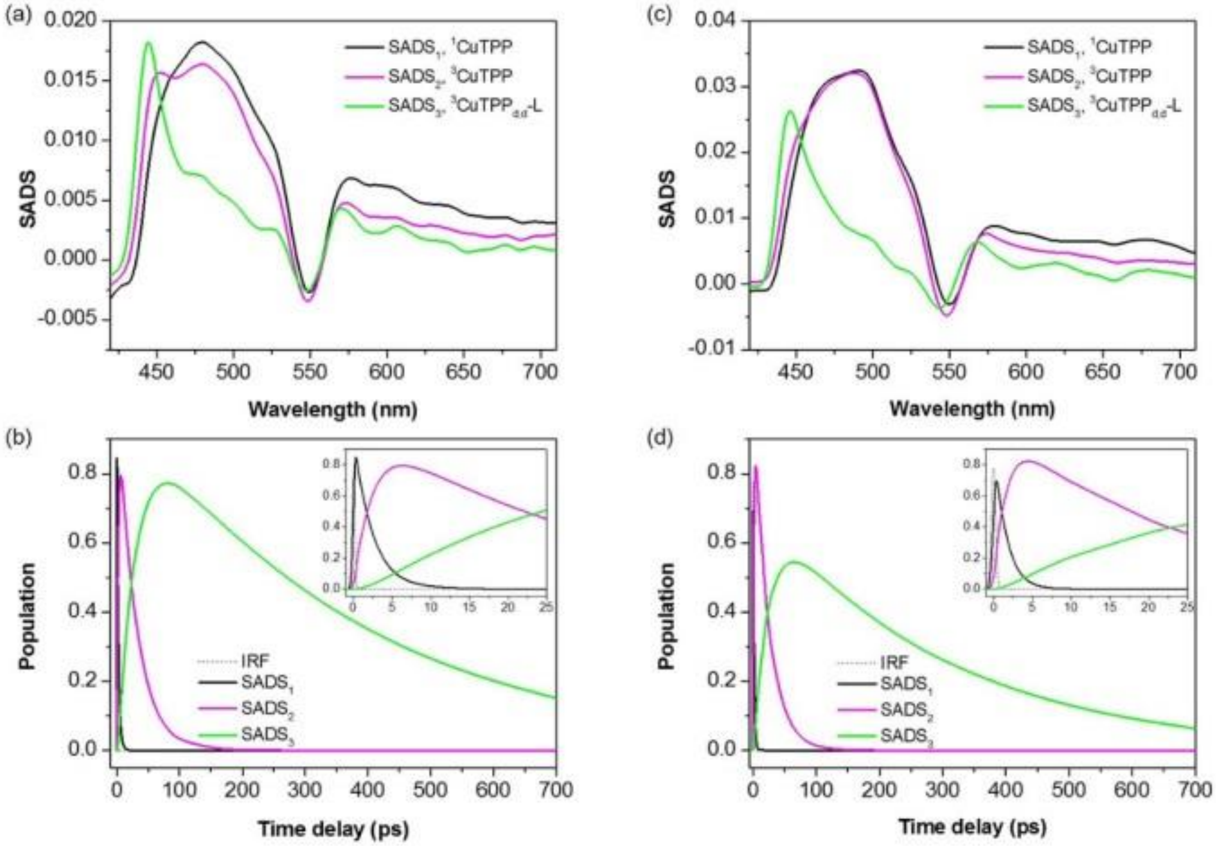

Figure S7. SADS of CuTPP in 1,4-dioxane (a-b) and THF (c-d) resulting from the target analysis of transient absorption data. The branching ratio of CT to $^2[d_{z2}, d_{x2-y2}]$ state is 0.04: 0.96 in 1,4-dioxane, and 0.32 : 0.68 in THF.

Sample	Solvent	Absorption peak (nm)					Emission peak (nm) // τ_f (ns)
		Soret	Q_y (1,0)	Q_y (0,0)	Q_x (1,0)	Q_x (0,0)	
H_2TPP	Benzene	419	514	549	591	647	654 // 9.8, 718 // (9.9)
	Pyridine	418	514	548	591	648	654 // 10.4, 718 // 10.7

Sample	Solvent	Absorption peak (nm)		
		Soret	Q (1,0)	Q (0,0)
CuTPP	Benzene	417	539	573
	2,6-lutidine	418	539	575
	Pyridine	422	545	588
	Piperidine	426	551	591
	THF	415	539	575
	1,4-dioxane	416	541	579

Table S1. Summary of absorption and emission bands for H_2TPP and CuTPP
